# Real-Time NURBS Interpolation under Multiple Constraints

**DOI:** 10.1155/2022/7492762

**Published:** 2022-05-17

**Authors:** Mingxing Nie, Yaping Wan, Aijun Zhou

**Affiliations:** School of Computer Science, University of South China, Hengyang 421001, China

## Abstract

NURBS interpolation is superior to traditional linear or circular interpolation in terms of code size, surface quality, and machining efficiency. However, with the increasing demands for high-accuracy and efficient machining, NURBS interpolation has faced a growing number of challenges. Many researchers are actively involved in this field with great interest. Due to the special form of NURBS curve, there is a nonlinear relationship between its curve and arc length; feed fluctuations and mechanical shocks which are caused during the interpolation process will seriously affect the surface accuracy and quality of machined parts. To solve these problems, a real-time NURBS interpolation is proposed under multiple constraints (RNIC) in this paper. First, the formulas of the constrained feedrate under geometric errors, kinematic constraints, drive constraints, and contour errors are given. Then, the two stages for the proposed interpolation are established. The former stage is offline preprocessing stage, which aims to quickly find feedrate sensitive areas (FSAs), while the latter online stage is the real-time interpolation, which is responsible for smoothing the velocity. In the preprocessing stage, we utilized FSA scan module and feedrate adjustment module to detect the FSAs and adjust the feedrate at the start/end of each subsegment by a bidirectional scanning algorithm. Each segment contains acceleration and deceleration (some contains uniform speed) stages, which can be well matched with the processing process of acceleration and deceleration. Finally, according to the proposed method and the adaptive speed adjustment method, the simulation of a “butterfly-shaped” NURBS curve using the S-shaped ACC/DEC algorithm is carried out, which verifies the reliability and effectiveness of the proposed algorithm.

## 1. Introduction

As one of the international industrial standards of CAD system, NURBS curves play an increasing role in the application fields of computer-aided geometric design (CAGD) with the wide application of this geometric model. Profiting from the ability of changing the shape of local contours and describing complex contours by low-order equations, NURBS interpolations have been increasingly developed in CNC machining. NURBS interpolation is superior to traditional linear and circular interpolation in terms of code size, surface quality, and machining efficiency. However, there are still some challenges in building a successful NURBS interpolator. First, the feedrate along the machining path may be limited by many factors including geometrical, kinematic, and axis dynamics constraints in large curvature areas; these constraints should be reasonably and smoothly adjusted to ensure good machining quality and accuracy. Second, abrupt curvature change will cause ACC/DEC and jerk out of tolerance. Last, there is no mathematical expression between the NURBS curve parameters and displacement, which inevitably leads to a fluctuation between the expected and actual feedrates.

In order to improve machining accuracy and efficiency, Yeh and Hsu [1] adaptively adjust the feedrate considering the constrained chord error, which improves the contour accuracy of parametric curve interpolation. Yong and Narayanaswami [2] proposed the concept of feedrate sensitive corners to confine acceleration and deceleration in offline processing. Nam and Liu [3, 4] further limited the effect of jerk on machining in NURBS interpolation. While these efforts are effective, many key constraints are overlooked; various advanced algorithms have been further developed by many researchers. For example, Sencer et al. [5] solved the analytical solution of feedrate in the form of cubic B-spline, but the calculation efficiency of this process is low. Sun et al. [6] proposed a feedrate scheduling method based on drive constraints. Xavier et al. [7] developed a velocity profile optimization method based on iterative calculation of intersection points with given constraints. However, this method ignores the influence of contour accuracy. Jia et al. [8] proposed a NURBS interpolator under kinetic characteristics and contour error constraints, in which the feedrate remained constant in most areas and only changed smoothly in areas with large curvature, but the production efficiency was degraded when utilizing a constant low feedrate without making full use of the motion performance in this method. Ma et al. [9] also considered, from the perspective of axis restriction, maintaining constant feedrate in the sensitive area and smooth transition in the non-sensitive area. Liu et al. [10] used a linear mathematical model to explore the relationship between feedrate and geometric constraints. Liu et al. [11] also developed a new feedrate optimization method under multiple constraints and finally fitted to the smooth spline curve. However, these two methods only considered the influence of geometric and axis dynamics errors. Zhong et al. [12] further limited the axial velocity, axial acceleration, jerk, and contour errors through a look-ahead module.

Moreover, to mitigate the feed fluctuation to further improve the machining accuracy, many methods have been proposed for NURBS interpolation. Liu et al. [13] proposed a NURBS interpolation using approximation errors, but the calculation coefficient polynomial equation is time-consuming. Liu et al. [14] proposed a highly accurate interpolation method by establishing an improved quartic equation related to the curve parameter increment to reduce the feedrate fluctuation caused by approximation errors. However, this method obtains a quartic equation by ignoring the higher-order term (order >4), which has limited adaptability. Ni et al. [15] proposed a round-off error-free feedrate scheduling method. However, this method limits the feedrate to meet the various constraints by increasing the interpolation time, which is not efficient. Song et al. [16] compensates the interpolation parameters in order to improve the calculation accuracy of the parameters for the next interpolation point. Jiang et al. [17] presented an iterative feedback interpolator that consists of estimator and corrector stages to restrain the feedrate fluctuation. Chen et al. [18] developed a NURBS interpolation method for deriving parametric curves based on Steffensen's iterative method to avoid unnecessary feedrate fluctuations. Ji et al. [19] calculated the interpolation point parameters through iterative correction to obtain the accurate position and reduce the federate fluctuates. However, these methods have difficulty guaranteeing convergence when using iterative methods. To reduce feedrate fluctuations, Wang et al. [20] developed a bi-interpolation algorithm using two second-order Taylor expansions in one interpolation cycle. However, the time cost of this algorithm is relatively large.

In addition, much effort has been made to develop feedrate optimization techniques, and many fruitful feedrate scheduling methods have been proposed. The feedrate profile of the linear acceleration and deceleration algorithm [21–23] is not smooth, and machine vibration will inevitably occur during the movement. In the early days, NURBS interpolation was realized by using the speed adaptive adjustment methods [1, 24], but these methods could not meet the tolerance limits of chord error, acceleration, and jerk. Du et al. [25] developed a complete S-shaped NURBS interpolation under confined jerk, acceleration limits, which adopted compensate on strategies to make the federate more continuous and the arc increment more precise. Lin et al. [26] used a look-ahead module to generate smooth feedrate profile to accommodate NURBS curve dynamics. Liu et al. [27] proposed the idea of NURBS curve segmentation to construct a new NURBS interpolator to improve contour error accuracy. Sun et al. [28] optimize short-line segmented machining using S-shaped ACC/DEC algorithm and time optimization function to obtain better machining accuracy and efficiency. Liu and Leng [14, 29] proposed a cubic polynomial NURBS interpolation. However, the cubic polynomial ACC/DEC algorithm is inefficient, and the jerk profile is discontinuous, which will impact the machine tool. Liu et al. [30] further improved and proposed the jerk-continuous NURBS interpolation method, which improved the contour machining accuracy and improved the low flexibility at small curvature sharp corners. However, these methods only consider the influence of the geometric characteristics of the curve and ignore the influence of the dynamic performance and contour error. Ni et al. [31] proposed a jerk-continuous NURBS interpolation. However, the computational load of this method is high, as the algorithm needs to deal with the coexistence of trigonometric functions and polynomial functions at the same time. Zhao et al. [32] applied a jerk-limited method to smooth the sharp corners of the final feedrate profile, which improved the feed correction polynomial to reduce the feed fluctuation. Furthermore, to address the problem of feedrate curve fluctuations in regions of large curvature, Su et al. [33] made full use of the local geometric information and adopted the method of sliding arc tube to optimize the target feedrate.

Traditional method is to subsegment the curve and then perform acceleration and deceleration processing based on local geometric information. In fact, when the arc length is less than the shortest requisite ACC/DEC distance, there are many types of ACC/DEC involved, and the processing is very complicated, which is not conducive to the realization of real-time interpolation. This article innovatively proposes a novel bidirectional scanning algorithm. For the curve subsegment with shorter arc length, directly process it at a uniform speed, discarding the complicated ACC/DEC process; for the long curve subsegment, readjust the acceleration end point or deceleration start point according to the acceleration or deceleration distance to meet the S-curve ACC/DEC requirements. This method is simple and efficient to process shorter curves, which is in line with the actual processing process of the special difficult-to-machine parts of the workpiece. Therefore, it can effectively improve the indicated processing quality of the processed part without increasing the hardware cost. Although the processing time is extended to a certain extent, generally speaking, because the arc length of the curve subsegment is shorter, it is meaningful to sacrifice a certain amount of time to obtain higher real-time performance and processing effect.

## 2. The Presented Interpolation Architecture

### 2.1. NURBS Parameter Discretization

NURBS curves can be represented by parameters as follows:

(1) $$\begin{array}{c} C\left(u\right)=\frac{\sum_{i=0}^{n}N_{i,p}\left(u\right)\omega_{i}p_{i}}{\sum_{i=0}^{n}N_{i,p}\left(u\right)\omega_{i}},\;u_{1}\leq u\leq u_{n+p+1}. \end{array}$$

where *i* is the index *i* = 0, 1,…, *n*; {**P***i*} is the set of control points; {*ωi*} is the set of weights; and {*Ni*, *p*(*u*)} is the set of *p*-order B-spline basic functions defined on the non-uniform knot vector **U**, *U*={*u*0,*u*1,…, *un*+*p*+1}. The *B*-spline basic function *Ni*, *p*(*u*) is calculated as

(2) $$\begin{array}{c} \left\{\begin{array}{c} N_{i,p}\left(u\right)=\frac{u-u_{i}}{u_{i+p}-u_{i}}N_{i,p-1}\left(u\right)+\frac{u_{i+p+1}-u}{u_{i+p+1}-u_{i+1}}N_{i+1,p-1}\left(u\right),\\ N_{i,0}\left(u\right)=\left\{\begin{array}{c} 1,\;u_{i}<u<u_{i+1},\\ 0,\;\text{otherwise}, \end{array}\right. \end{array}\right. \end{array}$$

The first-order and second-order derivatives of *B*-spline basic functions and NURBS curves are, respectively, given as

(3) $$\begin{array}{c} N_{i,p}^{\prime }\left(u\right)=\frac{p}{u_{i+p}-u_{i}}N_{i,p-1}\left(u\right)-\frac{p}{u_{i+p+1}-u_{i+1}}N_{i+1,p-1}\left(u\right). \end{array}$$

The first derivative and second derivative of *c*(*u*) can be calculated as

(4) $$\begin{array}{c} \left\{\begin{array}{c} C^{\prime }\left(u\right)=\frac{\sum_{i=0}^{n}N_{i,p}^{\prime }\left(u\right)\omega_{i}p_{i}-c\left(u\right)\cdot \sum_{i=0}^{n}N_{i,p}^{\prime }\omega_{i}}{\sum_{i=0}^{n}N_{i,p}\omega_{i}}\text{, }\\ C^{\prime \prime }\left(u\right)=\frac{\sum_{i=0}^{n}N_{i,p}^{\prime \prime }\left(u\right)\omega_{i}p_{i}-2c^{\prime }\left(u\right)\cdot \sum_{i=0}^{n}N_{i,p}^{\prime }\omega_{i}-c\left(u\right)\cdot \sum_{i=0}^{n}N_{i,p}^{\prime \prime }\omega_{i}}{\sum_{i=0}^{n}N_{i,p}\omega_{i}}, \end{array}\right. \end{array}$$

For a point *c*(*u*) on the NURBS curve, the curvature radius *ρ*(*u*) can be calculated by

(5) $$\begin{array}{c} \rho \left(u\right)=\frac{\left\|C^{\prime }{\left(u\right)}^{3}\right\|}{\left\|C^{\prime }\left(u\right)\times C^{\prime \prime }\left(u\right)\right\|}, \end{array}$$

where ‖·‖ stands for the Euclidean norm and × means the exterior product.

In this paper, the Adams-Bashforth-Moulton method (ABM) is adopted to discretize the parameter vector for NURBS interpolation. The ABM method is as follows:

(6) $$\begin{array}{c} \left\{\begin{array}{c} u_{m+1}=u_{m}+\frac{T}{24}\left(55f_{k}-59f_{k-1}+37f_{k-2}-9f_{k-3}\right),\\ f_{k}=\left|\frac{\text{d}u}{\text{d}t}\right|t=t_{m}=\frac{v\left(u_{m}\right)}{\left\|C^{\prime }\left(u_{m}\right)\right\|}=\frac{v\left(u_{m}\right)}{\sqrt{{\left(dC_{x}\left(u_{m}\right)/du_{m}\right)}^{2}+{\left(dC_{y}\left(u_{m}\right)/du_{m}\right)}^{2}+{\left(dC_{z}\left(u_{m}\right)/du_{m}\right)}^{2}}}, \end{array}\right. \end{array}$$

where *v*(*u*_*m*_), *T*, and *C*′(*u*_*m*_) are the velocity, the interpolation period, and the first derivatives of the NURBS curve; *C*_*x*_(*u*_*m*_), *C*_*y*_(*u*_*m*_), and *C*_*z*_(*u*_*m*_) denote the parameter curve; *u*_*m*_ corresponds to each axis component of the interpolation point *C*(*u*_*m*_) on the curve.

The NURBS interpolation process is continuously affected by geometric errors, motion errors, drive errors, and contour errors. Aiming at the above problems, in the preprocessing stage, under the premise of considering multiple constraints, a real-time NURBS interpolation under multiple constraints (RNIC) is proposed, which can accurately obtain the constrained interpolation feedrate and optimal interpolation points.

### 2.2. Architecture of RNIC NURBS Interpolation

**Figure 1:**
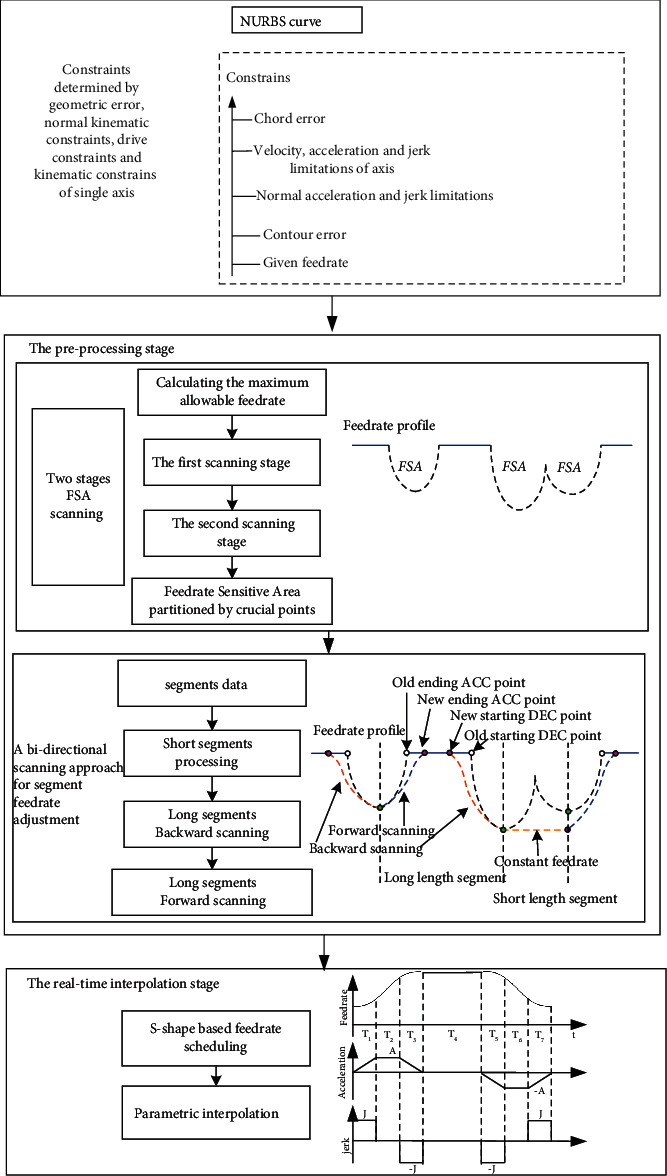
The structure of the FSA NURBS interpolator.

A systematic NURBS interpolator with smoothing feedrate at the FSA under multiple constraints is developed to enhance the interpolator performance with respect to the machining precision and machining quality. The overall structure of the interpolator is shown in Figure 1. The interpolator has two stages, namely, the preprocessing stage and the real-time interpolation stage. The former preprocessing stage aims to find the FSA quickly, while the latter real-time interpolation implements velocity smoothing and generation of interpolated toolpath points.

The interpolator comprehensively considers various constraints, breaking through the geometric constraints, kinematic feature constraints, and machine performance constraints of NURBS interpolation, including various constraints such as chord error, velocity, normal acceleration and jerk, contour error, and command feedrate. There are two modules in the preprocessing stage, namely, the FSA scan module and the segment feedrate adjustment module, which are used to obtain the geometric features required by the NURBS curve as the input for the next step of real-time interpolation.

The goal of the FSA scan module is to efficiently obtain FSAs of the NURBS curve by introducing the first FSA scan algorithm, then find the critical point in each FSA in the second FSA scan algorithm and divide the FSA curve blocks into subsegments, and further get more detailed information about the geometric features of subsegments. The goal of the segment feedrate adjustment module is to optimize the feedrate profile for segments data generated by the former FSA scan module, which includes short segments processing, long segments backward scanning, and long segments forward scanning. During short segments processing, update the feedrate of the start/end point at the beginning and end of the segment to the same smaller speed, and then keep the entire short segment interpolation speed constant; during long segments processing, use backward and forward directions. In the forward scanning process, in order to simplify the processing flow, first set the start/end speed of the beginning and end to the smaller speed, and then obtain the actual maximum allowable feedrate of each segment; in the backward scanning process, the starting point of deceleration for a long segment is obtained.

In the real-time interpolation stage, it is mainly to obtain the feedrate and calculate the precise coordinates of the next interpolation point. The s-shape based feedrate scheduling is adopted to achieve smooth feedrate profile which can greatly improve the accuracy of interpolation.

## 3. Scheduling of the FSA under Multiple Constraints

This paragraph describes the scheduling method of FSA. First, determine the allowable feedrate on the NURBS curve under the constraints of geometric characteristics, dynamic characteristics, etc., and give its corresponding mathematical function formula. Then, the FSAs are detected quickly with a two-stage scanning method, which employs FSA scan and feedrate adjustment modules. The goal of the FSA scan module is to quickly find the FSA, including the maximum allowable feedrate calculation, the first scanning stage, the second scanning stage, and the FSA partition. The goal of the FSA feedrate adjustment is to optimize the start/end feedrate of the acceleration/deceleration phase, which includes intersection processing, backward scanning, and forward scanning.

### 3.1. Allowable Feedrate under Multiple Constraints

#### 3.1.1. Allowable Feedrate for Chord Error Constraint

**Figure 2:**
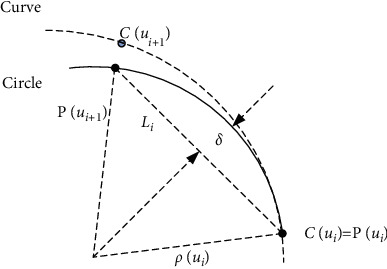
The chord error representation.

Considering the chord $\bar{C\left(u_{i}\right),C\left(u_{i+1}\right)}$ is a tiny segment in Figure 2, the arc approximation method can be used to calculate the chord error. In Figure 2, *P*(*u*_*i*_) is a point on the circle, *C*(*u*_*i*_) is a point on the curve, *P*(*u*_*i*_) and *C*(*u*_*i*_) are the coincident interpolation points, and the parameter vectors are both *u*_*i*_. *P*(*u*_*i*+1_), *C*(*u*_*i*+1_) are the next estimated interpolation points, and their parameter vectors are both *u*_*i*+1_.

Considering $L_{i}=\bar{C\left(u_{i}\right),C\left(u_{i+1}\right)}$, we can get ‖*P*(*u*_*i*+1_) − *P*(*u*_*i*_)‖=*L*_*i*_, the approximated feedrate *v*(*u*_*i*_)=*L*_*i*_/*T*, and the formula for calculating chord error *δ* is

(7) $$\begin{array}{c} \delta =\rho \left(u_{i}\right)-\sqrt{\rho {\left(u_{i}\right)}^{2}-{\left(\frac{v\left(u_{i}\right)T}{2}\right)}^{2}}. \end{array}$$

*ρ*(*u*_*i*_) is the curvature radius of the interpolated point *C*(*u*_*i*_), and *v*(*u*_*i*_) is the velocity during the sampling time *T*. The allowable feedrate *v*_*c*1_(*ρ*(*u*_*i*_)) of the interpolated point *C*(*u*_*i*_) for the set maximum chord error *δ*_max_ is

(8) $$\begin{array}{c} v_{c1}\left(\rho \left(u_{i}\right)\right)=\frac{2\sqrt{2\rho \left(u_{i}\right)\delta_{\max}-\delta_{\max}^{2}}}{T}. \end{array}$$

#### 3.1.2. Allowable Feedrate for the Normal Acceleration and Normal Jerk Constraints

Acceleration is the rate at which the speed and direction of an object are changing; it can be broken into its tangent and normal components. Here we first consider the normal acceleration; it can be expressed as

(9) $$\begin{array}{c} a_{n}=\frac{v^{2}}{\rho }. \end{array}$$

Therefore, the allowable feedrate *v*_*c*2_(*ρ*(*u*_*i*_)) by the normal acceleration constraint is given as

(10) $$\begin{array}{c} v_{c2}\left(\rho \left(u_{i}\right)\right)=\sqrt{\rho \left(u_{i}\right)a_{n,\max}}, \end{array}$$

where *a*_*n*,max_ is the maximum normal acceleration.

The normal jerk is calculated as

(11) $$\begin{array}{c} \left\{\begin{array}{c} \Delta a_{n}=\left\|a_{n+}-a_{n-}\right\|=2a_{n}\sin\left(\frac{\Delta \varphi }{2}\right)=2\frac{v^{2}}{\rho }\sin\left(\frac{\Delta \varphi }{2}\right),\\ \Delta t=\frac{\rho \Delta \varphi }{v},\\ j_{n}=\underset{\Delta t⟶0}{\lim}\frac{\Delta a_{n}}{\Delta t}=\underset{\Delta \varphi ⟶0}{\lim}\frac{2v^{2}/\rho \sin\left(\Delta \varphi /2\right)}{\rho \Delta \varphi /v}=\frac{v^{3}}{\rho^{2}}. \end{array}\right. \end{array}$$

Hence, the allowable feedrate *v*_*c*3_(*ρ*(*u*_*i*_)) for the normal jerk constrained can be derived as

(12) $$\begin{array}{c} v_{c3}\left(\rho \left(u_{i}\right)\right)=\sqrt[{3}]{\rho {\left(u_{i}\right)}^{2}j_{n,\max}}. \end{array}$$

where *j*_*n*,max_ is the maximum normal jerk.

#### 3.1.3. Allowable Feedrate for the Contour Error Constraint

The contour error directly affects the machining effect of the workpiece. For this reason, we include the contour error constraint in the preprocessor process to effectively limit the contour error from the source. Accurate estimation of contour errors has a significant impact on the quality of contour control. The contour error should satisfy the following formula:

(13) $$\begin{array}{c} \varepsilon <\varepsilon_{\lim}, \end{array}$$

where *ε*_lim_ is the given contour error.

The servo-lag induced contour error in real machining is considered as an additional constraint accompanied by the geometric and drive constraints during feedrate scheduling, so that the contour error can be bounded from the source. According to the literature [8], the feedrate constraint formula under the limitation of contour error is introduced as follows:

(14) $$\begin{array}{c} v_{c4}\left(\rho \left(u_{i}\right)\right)=\frac{\sqrt{2\rho \left(u_{i}\right)\varepsilon_{\max}}}{T}, \end{array}$$

Thus, the constrained interpolation feedrate *v*_cons_(*u*) can be obtained as

(15) $$\begin{array}{c} v_{\text{con}}\left(u_{i}\right)=\min\left(v_{c1}\left(\rho \left(u_{i}\right)\right),v_{c2}\left(\rho \left(u_{i}\right)\right),v_{c3}\left(\rho \left(u_{i}\right)\right),v_{c4}\left(\rho \left(u_{i}\right)\right),V_{F}\right). \end{array}$$

*v*_con_(*u*_*i*_) is the allowable feedrate under multiple constraints. It can be seen that the constrained feedrate is only related to the curvature of the point. However, due to the continuous influence of curve curvature fluctuation, NURBS interpolation will still be affected by the tolerance of tangential acceleration and jerk in the large curvature part. Then, a novel curve subsegmentation method was proposed during the preprocessing stage using a two-stage FSA scan method to obtain sufficient geometric characteristics of the curve. Compared with the existing methods, this method not only considers the optimal speed under various constraints, but also uses the preprocessing process to obtain enough curve geometric characteristic information without adding additional hardware.

### 3.2. The Preprocessing Stage

#### 3.2.1. Curve Splitting at Breakpoints

During acceleration and deceleration on real-time interpolation of NURBS curves, the remaining curve distance needs to be calculated. The adaptive Simpson method can be used to calculate the length of the subsegment of the curve [8, 27]. But if there are breakpoints that appear to be discontinuous, C1 continuity is not satisfied which is a necessary condition for adaptive Simpson method. Therefore, the key is to find the breakpoints on the curve and segment the curve with the breakpoint as the boundary.

There are roughly two types of breakpoints [3]. The first type is the points of C0 continuity. The second type is the points with C1 continuity when there are multiple corresponding control points.

Then, the NURBS curve can be segmented into blocks according to these two kinds of breakpoints, and the buffer {[*u*_*s*_^block*i*^, *u*_*e*_^block*i*^]} represented as the parameter interval of i-th block.

#### 3.2.2. The Two-Stage FSA Scan

Although NURBS curves are roughly divided into blocks according to breakpoints, the interpolation of each block will continue to be affected by the change of curve curvature. In order to realize smoothness interpolation, ACC/DEC feedrate profile needs to be adopted in the interpolation. The more geometrical characteristic obtained, the better the acceleration and deceleration effect.

This paper creatively proposes a two-stage feedrate sensitive area (FSA) scanning method that can efficiently obtain sufficient geometrical characteristic without adding extra burden to real-time interpolation module. FSA represents a special area of the curve, which reflects the area where the allowable feedrate on the curve is less than the commanded feedrate.

The first stage of an FSA scanning method is to obtain the FSAs of the curve, and the second stage is to get more detailed information of FSAs, such as the parameter of the start/end point, critical point, and the corresponding constrained feedrate of the FSAs.

In the first stage scanning, the input data is the curve block parameter interval [*u*_*s*_^block^, *u*_*e*_^block^] which is the output of Ssection 3.1. Firstly, the parameter interval is discretized into a set of elements {*u*_*i*_} by step size Δ*u*. Then, the constrained feedrate *v*_con_(*u*_*i*_) of each point *C*(*u*_*i*_) is calculated according to (15). If *v*_con_(*u*_*i*_) < *v*_*F*_, then the point *C*(*u*_*i*_) belongs to the feedrate sensitive area (FSA); otherwise, it belongs to the feedrate insensitive area (FIA).

Therefore, the points of the curve block can be classified to FSA and FIA datasets through the first stage scanning. If the FSA set is not empty, it indicates that there are some feedrate sensitive areas in the curve block, and the constrained feedrates of the points in FSA are lower than the command feedrate. If the FIA set is not empty, it indicates that there are some feedrate insensitive areas in the curve block, and the constrained feedrates of the points in the FIA can reach the command value *v*_*F*_.

The step size Δ*u* is important in the first stage scanning; the larger the step size is, the fewer the points are. If the step size is very large, some FSAs will be lost, and if the step size is too small, the processing time will be increased. In actual processing, the step size shall be flexibly selected according to experience and curve characteristics.

After that, the second scanning stage is performed; the input data is the FSA and FIA datasets of the first stage scanning. In this stage, the curve blocks are further divided into subsegment, and it is necessary to obtain the geometrical characteristics information, such as the start/end point of the FSA.

Suppose there are four points *C*(*u*_*a*_), *C*(*u*_*b*_), *C*(*u*_*i*_), *C*(*u*_*j*_) on a curve block, where the parameters *u*_*a*_, *u*_*b*_ ∈ FGA_*m*_ , and *u*_*i*_, *u*_*j*_ ∈ FIA_*m*_, *C*(*u*_*a*_) and *C*(*u*_*b*_) are the adjacent points. If *u*_*a*_ < *u*_*i*_ < *u*_*j*_ < *u*_*b*_, there must exist a feedrate sensitive area in the parameter interval [*u*_*a*_, *u*_*b*_]. So the second stage scanning is carried out for the detailed information of the FSA.

In the second stage scanning, the parameter interval [*u*_*a*_, *u*_*b*_] is discretized into a set of elements {*u*_*i*_}_*i*=1…*n*_ by step size Δ*u*′, and the corresponding curve is divided into *n* points. The constrained velocity of each point *C*(*u*_*i*_) is calculated according to equation (6). If *v*_con_(*u*_*i*−1_)==*v*_*F*_ and *v*_*con*_(*u*_*i*_) < *v*_*F*_, it means that the velocity of the previous point *C*(*u*_*i*−1_) is exactly equal to the command velocity *v*_*F*_ and the velocity of the current point *C*(*u*_*i*_) is lower than the command velocity *v*_*F*_, and the current point *C*(*u*_*i*_) is considered to be the start point of the FSA; if *v*_*con*_(*u*_*i*_) < *v*_*F*_ and *v*_*con*_(*u*_*i*+1_)==*v*_*F*_, it means that the velocity of the next point *C*(*u*_*i*+1_) is exactly equal to the command velocity *v*_*F*_ and the velocity of the current point *C*(*u*_*i*_) is lower than the command velocity *v*_*F*_; the current point *C*(*u*_*i*_) is considered to be the end point of the FSA; if the velocity of point *C*(*u*_*i*_) is the point with the lowest velocity of all points in the parameter interval [*u*_*a*_, *u*_*b*_], then point *C*(*u*_*i*_) is considered to be the critical point of the FSA which has the lowest velocity and minimum radius of curvature.

So far, the detailed geometrical feature information of the FSA is obtained, such as the start/end point of the FSA. So the quaternion tuple [*u*_*s*_, *u*_*c*_, *v*_*c*_, *u*_*e*_] corresponding to a FSA is got with the parameter *u*_*s*_ of the start point, the parameter *u*_*c*_ of the critical point and its constrained feedrate *v*_*c*_, and the parameter *u*_*e*_ of the end point. To obtain accurate parameter of the critical point, a smaller step size Δ*u*′ in the second stage scanning is required, which should be much smaller than the step size Δ*u* in the first stage scanning.

The critical point has minimum radius of curvature and lowest feedrate in an FSA. Therefore, the critical point can be regarded as the start/end point of the acceleration/deceleration process in ACC/DEC feedrate profile. Between the two adjacent critical points, there is only one acceleration process and one deceleration according to the characteristics of NURBS curve. Hence, the curve block can be divided into several subsegments by critical points. This will greatly simplify feedrate planning for the feedrate profile of each subsegment has only one acceleration and one deceleration process.

**Figure 3:**
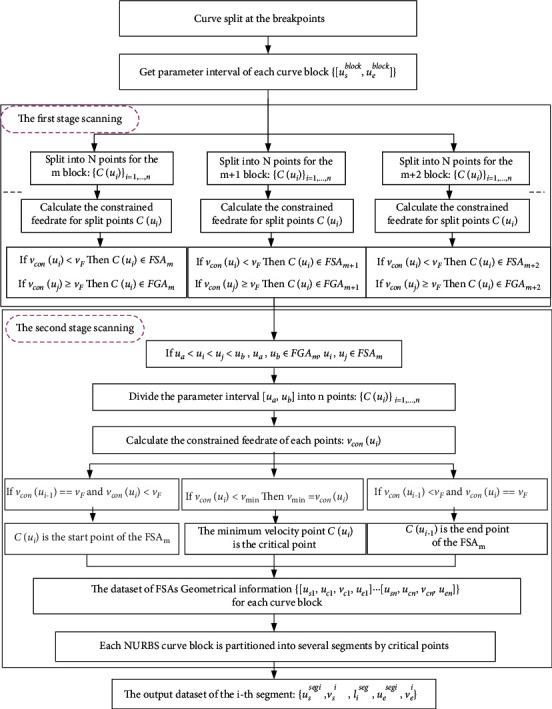
The flow chart of the two-stage FSA scan.

After the seconde stage scanning, The NURBS curve block can be partitioned into subsegments by critical points with the datasets {*u*_*s*_^*segi*^, *v*_*s*_^*i*^, *l*_*i*_^*seg*^, *u*_*e*_^*segi*^, *v*_*e*_^*i*^} , where *u*_*s*_^*segi*^ , *u*_*e*_^*segi*^ are the parameters of the start point and end point, with the corresponding feedrate *v*_*s*_ and *v*_*e*_, and *l*_*i*_^*seg*^ is the arc length for the parameter interval [*u*_*s*_^*segi*^, *u*_*e*_^*segi*^], which can be calculated by the adaptive simpson method [8, 27]. The flow chart of the two-stage scanning method is shown in Figure 3.

### 3.3. A Bidirectional Scanning Approach for the Subsegments

The curve is divided into subsegments according to the critical point following the above method. If the arc length is not long enough, the feedrate cannot reach the command feedrate before deceleration. In addition, when the arc length is too short, even if the feedrate keeps accelerating or decelerating, it cannot reach the end velocity before reaching the endpoint of the subsegment. To deal with the short curve segments, there are seventeen types of feedrate profiles in [25], leading to the complex and time consume processing. In another paper, the authors proposed a new method that the scheduled feedrate keeps constant at most areas [8]; this method greatly simplifies the processing process, but the machining efficiency is also greatly reduced.

For dataset {*u*_*s*_^*segi*^, *v*_*s*_^*i*^, *l*_*i*_^*seg*^, *u*_*e*_^*segi*^, *v*_*e*_^*i*^} of the i-th subsegment, the requirement ACC/DEC displacements can be deduced as follows:

(16) $$\begin{array}{c} S_{\text{acc}/\text{dec}}^{\text{req}}\left(v_{s}^{i},v_{e}^{i}\right)=S_{\text{acc}}^{\text{req}}\left(v_{s}^{i},v_{F}\right)+S_{\text{dec}}^{\text{req}}\left(V_{\max},v_{e}^{i}\right)+nTv_{F}, \end{array}$$

where *S*_acc/dec_^req^(*v*_*s*_^*i*^, *v*_*e*_^*i*^) is the requirement ACC/DEC displacements, *S*_acc_^req^(*v*_*s*_^*i*^, *V*_max_) is the acceleration displacements, *S*_dec_^req^(*V*_max_, *v*_*e*_^*i*^) is the deceleration displacements, and *v*_*F*_ is the command feedrate. There will exits a constant feedrate profile between acceleration and deceleration when *n* > 0 for. The minimum required ACC/DEC displacement is obtained if *n* = 0 as follows:

(17) $$\begin{array}{c} S_{\text{acc}/\text{dec}}^{\min}\left(v_{s}^{i},v_{e}^{i}\right)=S_{\text{acc}}^{\text{req}}\left(v_{s}^{i},v_{F}\right)+S_{\text{dec}}^{\text{req}}\left(v_{F},v_{e}^{i}\right). \end{array}$$

And if

(18) $$\begin{array}{c} l_{i}^{\text{seg}}<S_{\text{acc}/\text{dec}}^{\min}\left(v_{s}^{i},v_{e}^{i}\right), \end{array}$$

it means that the arc length *l*_*i*_^*seg*^ is shorter than minimum required ACC/DEC displacement, and the feedrate profile of the segment cannot achieve the command federate *v*_*F*_ during the ACC/DEC procedure. If the arc length *l*_*i*_^*seg*^ is longer than minimum required ACC/DEC displacement, the feedrate profile can accelerating from *v*_*s*_^*i*^ to *v*_*F*_ and decelerating from *v*_*F*_ to *v*_*e*_^*i*^. In this case, the start point of the deceleration should be decided precisely for the real-time interpolation.

Therefore, we propose a bidirectional scanning method to further process the subsegment curve which consists of two stages: (1) forward scanning; (2) backward scanning.

The forward scanning is to reduce the types of feedrate profiles and improve time efficiency for short subsegment. First, the feedrate of the start/end point of the subsegment is set to be the same value, so that all subsegment can be regarded as the superposition of acceleration and mirror deceleration processes. Without loss of generality, the acceleration distance from the start point to the end point is exactly half of the arc length of the subsegment; that is, the acceleration from the start point to the end point just reaches the actual maximum feedrate. This not only takes into account the efficiency, but all short subsegments can use the same method to simplify the processing method. After forward scanning, each subsegment updates feedrate of the start/end point and gets the maximum allowable feedrate *V*_max_ that can actually be achieved and obtains the output dataset {*u*_*s*_^segi^, *v*_*s*_^*i*^, *l*_*i*_^seg^, *V*_max_, *u*_*e*_^segi^, *v*_*e*_^*i*^}.

The backward scanning is to obtain the deceleration start point of each subsegment. For the long subsegment, the deceleration feedrate profile is decelerating from the command velocity to the velocity of the end point, while the short subsegment is decelerating from the actual maximum velocity to the velocity of the end point. The deceleration velocity profile is just the opposite of the acceleration velocity profile. The deceleration process can be regarded as the inverse process of acceleration, so as to get the start point of the deceleration. Through the backward scanning, the deceleration starting point can be found and gets the output dataset {*u*_*s*_^segi^, *v*_*s*_^*i*^, *l*_*i*_^seg^, *V*_max_, *u*_dec_, *u*_*e*_^segi^, *v*_*e*_^*i*^}, where *u*_dec_ is the parameter of the start point for the deceleration process.

The bidirectional scanning algorithm is mainly to adapt to the changing curvature characteristics of the NURBS curve. It uses the forward scanning to handle the feedrate at the start and end of the curve segment, as well as the actual maximum feedrate. It uses the backward scanning to get the deceleration starting point. However, the limitation of the bidirectional scanning algorithm is that if the velocities of the start and end points are different, the velocities at the convergence point of forward and backward interpolation need to be calculated, and the acceleration needs to be readjusted to make the velocity transition smoothly. In this paper, the feedrate of the start and end point of the subsegment is set to be the same value, so that all subsegments can be regarded as the superposition of acceleration and mirror deceleration processes.

The pseudocode representation of the forward scanning and backward algorithm are shown in Tables 1 and 2, and the specific steps are as follows.

- Forward scanning:
- Step 1. Let i = 0, i is the counter;
- Step 2. Get the arc length *l*_*i*_^seg^, the starting feedrate *v*_*s*_^*i*^, and the ending feedrate *v*_*e*_^*i*^ of the i-th subsegment;
- Step 3. Calculate the minimum required ACC/DEC displacement *S*_*seg*_^min^(*v*_*s*_^*i*^, *v*_*e*_^*i*^) of the i-th subsegment accelerating from *v*_*s*_^*i*^ to *v*_F_ (command feedrate) and decelerating from *v*_F_ to *v*_*e*_^*i*^ under an S-shape ACC/DEC feedrate profile described in reference 26 with acceleration and jerk of *A* and *J*;
- Step 4. If *l*_*i*_^*seg*^ < *S*_*seg*_^min^(*v*_*s*_^*i*^, *v*_*e*_^*i*^), go to Step 5; otherwise, set *V*_max_=*v*_*F*_, go to Step 7.
- Step 5. Update *v*_*s*_^*i*^ and *v*_*e*_^*i*^ by equation *v*_*s*_^*i*^=*v*_*e*_^*i*^=min(*v*_*s*_^*i*^, *v*_*e*_^*i*^). If the velocity at the starting/ending point of the previous subsegment is updated, the velocity at the starting/ending point of the adjacent subsegment should be updated to a lower velocity by an iterative method.
- Step 6. Recalculate the new maximum achievable feedrate *V*_max−i_^*act*^ to satisfy *S*(*v*_*s*_^*i*^, *V*_max−*i*_^act^)=*l*_*i*_^*se*g^/2 that accelerated from *v*_*s*_^*i*^ to *V*_max−*i*_^act^ and the displacement is half of the subsegment arc length, set *V*_max_=*V*_max−*i*_^act^.
- Step 7. If i< N, N represents the number of subsegments, set i = i+1; then go to Step 2; otherwise, end the program.

(B) Backward scanning:
  Step 1. Set i = N; N represents the number of subsegments.
  Step 2. Get the arc length *l*_*i*_^*seg*^, maximum feedrate *V*_max_, and the end feedrate *v*_*e*_^*i*^ of the i-th subsegment from the forward scanning output.
  Step 3. If *V*_max_==*v*_*F*_ (command Feedrate), take the velocity of the ending point as the starting velocity, and *v*_*F*_ as ending velocity. The deceleration start point *u*_dec_^*i*^ calculated reversely from *u*_*e*_^*i*^ is the deceleration start point under S-shape ACC/DEC method described in reference 26; if *V*_max_ < *v*_*F*_, take the velocity of the ending point as starting velocity, and *V*_max_ as ending velocity, *l*_*seg*_^*i*^/2 as the displacement. Calculate the deceleration start point *u*_dec_^*i*^ from *u*_*e*_^*i*^ reversely.
  Step 4. If *i* = 1, program ends; otherwise, set *i* = *i*−1, and go to Step 2.

**Figure 4:**
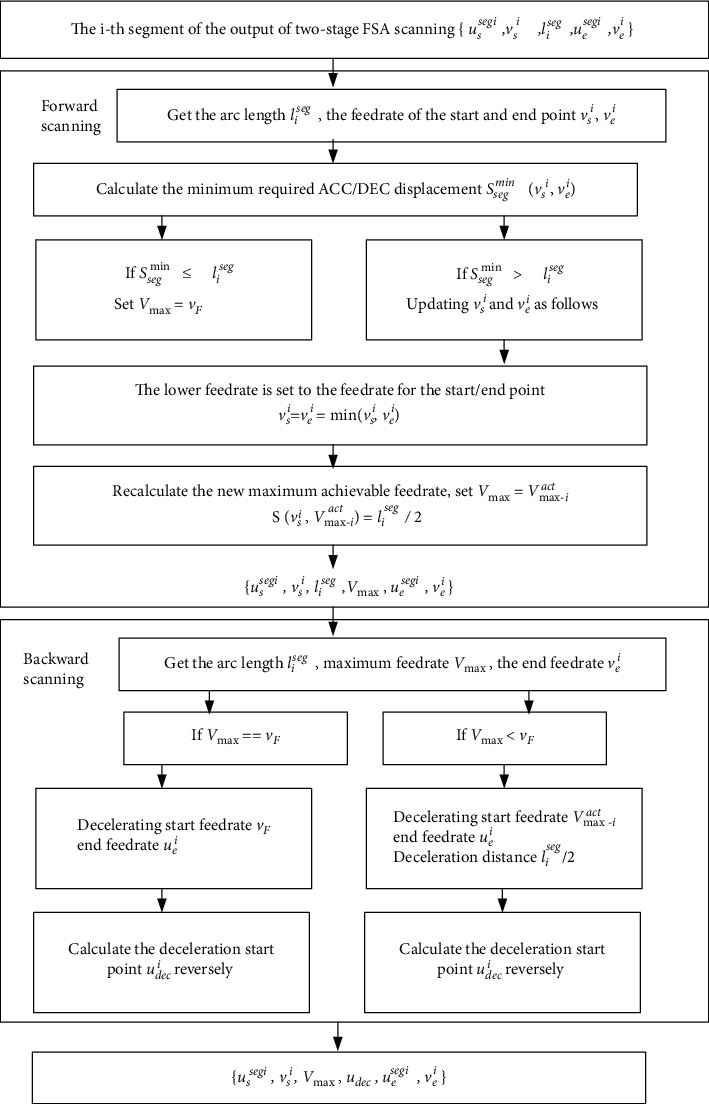
The bidirectional scanning method.

After bidirectional scanning, the dataset of the final preinterpolation is obtained as {*u*_*s*_^*segi*^, *v*_*s*_^*i*^, *V*_max_, *u*_dec_, *u*_*e*_^*segi*^, *v*_*e*_^*i*^}, where *u*_*s*_^*segi*^ and *u*_*e*_^*segi*^ are the parameters of the start/end point for the *i*-th subsegment, *v*_*s*_^*i*^ and *v*_*e*_^*i*^ are the corresponding feedrate, *V*_max_ is the maximum achieved feedrate, and *u*_dec_ is the parameter of the start point of deceleration. The flow chart of the bidirectional scanning approach is shown in Figure 4.

## 4. Simulation and Results

**Figure 5:**
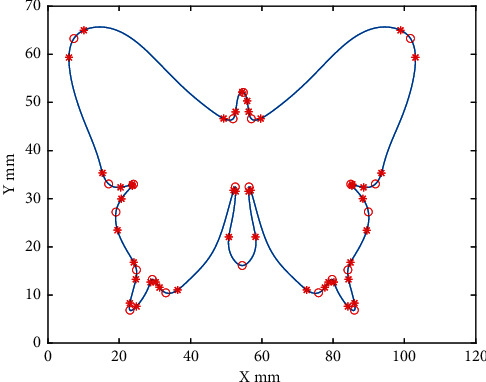
The butterfly-shaped curve.

The proposed NURBS interpolator is simulated on an Intel Core i7-8565U 1.8 GHz laptop. The butterfly-shaped curve is used for the proposed method shown in Figure 5. The proposed real-time NURBS interpolation under multiple constraints (RNIC) and adaptive feedrate interpolation (AFI) are adopted for comparison. The two methods are implemented for the butterfly-shaped curves, and the parameters are shown in Tables 3 and 4.

**Figure 6:**
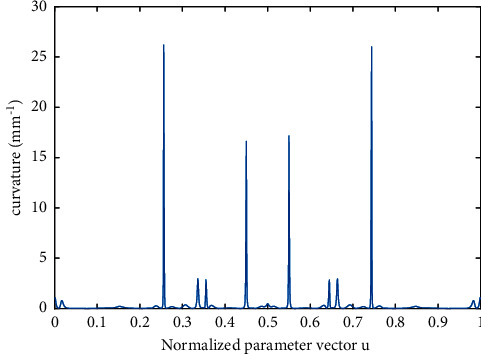
The curvature of the butterfly-shaped curve.

The curvature of the test curve is shown Figure 6. The test curve has high curvature and low curvature regions. In the low curvature area, due to the constraint of curvature, the velocity will not be able to feed at the command velocity, which can be a good verification of our proposed method.

Both preprocessing and online interpolation routines are developed in C. Additionally, we use MATLAB software to quantitatively analyze the actual velocity, tangential acceleration, and tangential jerk, which can be expressed as follows:

(19) $$\begin{array}{c} \left\{\begin{array}{c} v_{\text{actual},i}=\frac{\left|C\left(u_{i+1}\right)-C\left(u_{i}\right)\right|}{T},\\ a_{\text{actual},i}=\frac{v_{i+1}-v_{i}}{T},\\ j_{\text{actual},i}=\frac{a_{i+1}-a_{i}}{T}. \end{array}\right. \end{array}$$

Figure 5 shows FSAs detected by the proposed scheme (RNIC) indicated by asterisk and hollow circle, where the asterisk marked points are the starting/ending point of a FSA, and the hollow circle marked points are the minimum velocity point of the FSAs, called the critical points. The NURBS curve block can be partitioned into subsegments by critical points. All information of segments for the test curve is listed in Table 5. There are 21 segments on the test curve, where U_start and U_end are the parameters of starting/ending point of segment, and V_start and V_end are the velocity of starting/ending point of the segment. Required arc length is the minimum length for the complete S curve acc/dec profile from U_start to U_end at the test curve. Acture arclength is the length of the curve from the U_start to U_end at the test curve. The complete S curve profile field shows that the curve segment can complete a complete acceleration and deceleration process when the value is 1, or cannot complete when the value is 0.

**Figure 7:**
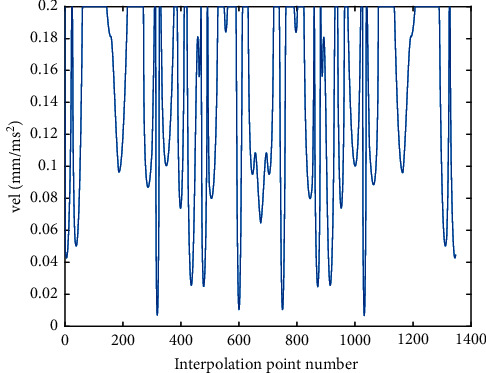
The velocity profile of the butterfly-shaped NURBS curve by AFI.

**Figure 8:**
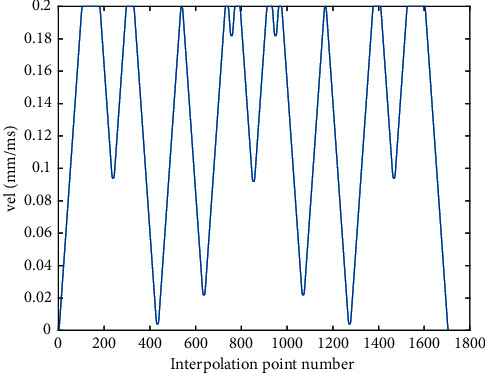
The velocity profile of the butterfly-shaped NURBS curve by RNIC.

**Figure 9:**
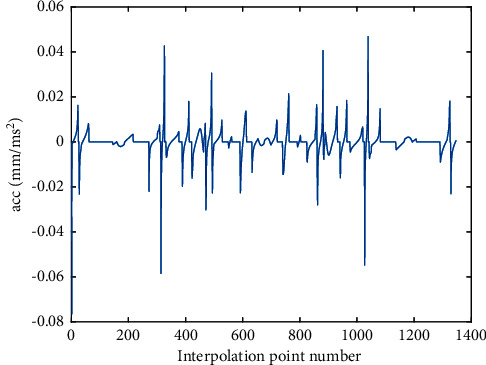
The ACC profile of butterfly-shaped NURBS curve by AFI.

**Figure 10:**
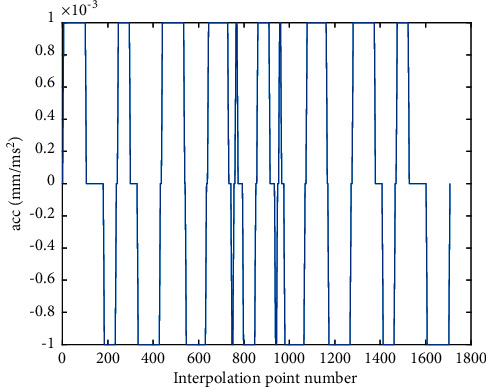
The ACC profile of butterfly-shaped NURBS curve by RNIC.

**Figure 11:**
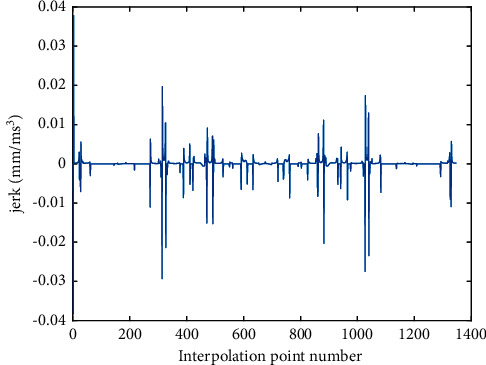
Jerk profile of butterfly-shaped NURBS curve by AFI.

**Figure 12:**
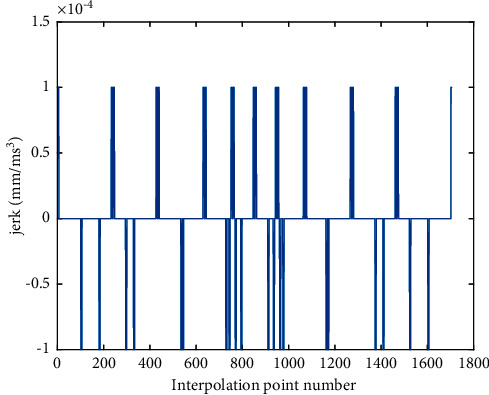
Jerk profile of butterfly-shaped NURBS curve by RNIC.

The optimized velocity profile by two methods is shown in Figures 7 and 8, and the corresponding tangential acceleration/tangential jerk profiles are presented in Figures 9–12. As depicted in Figures 7 and 8, the feedrate is under the command feedrate by two methods. However, as shown in Figure 7, the starting speed of the feedrate profile in AFI method is rising directly from 0 to the command feedrate, which will cause a large feedrate vibration and affects the processing quality. A similar situation exists at the ending point in Figure 7. In the AFI method, the feedrate can meet the contour error limit adapted by the chord error, but the tangential acceleration exceeds the given tolerance at most of the time as shown in Figure 9. The maximum tangential acceleration is 0.0076 mm/s^2^ by AFI method, as shown in Table 5, which is seven times more than the maximum allowable value. At the same time, the fluctuation of acceleration also causes the tangential jerk to exceed the maximum allowable value. The maximum tangential jerk is 0.0382 mm/s^3^ by AFI, as shown in Table 6, which is 380 times more than the maximum allowable value.

Compared with the AFI method, As shown in Figure 8, the initial speed of the feedrate profile in the proposed RNIC method starts at zero and then accelerates to the command speed. Correspondingly, at the end of the feedrate profile, the speed decelerates to zero. In the feedrate scheduling process, the feedrate is optimized under multiple constraints. Therefore, from Figures 10 and 12, the tangential acceleration and tangential jerk by the FSA method can met system requirements; all tangential kinematic constraints of the proposed method are satisfied perfectly as shown in Table 6. Thus, the smoothness of the speed of the interpolation process is ensured.

The proposed RNIC method comprehensively considers the constraint of chord error, normal acceleration, normal jerk, and contour error in the preprocessing stage and directly introduces S-shaped ACC/DEC to the curve segments in the real-time interpolation. Compared with the AFI method, the proposed RNIC method adds a preprocessing module, which can realize the optimal feed under various constraints. It can be seen from the simulation results that the former has a greater improvement in speed smoothness than the latter. From the perspective of real-time processing, the former arranges non-real-time tasks to be completed in the preprocessing stage which can greatly reduce the working load of the real-time interpolation module, so that high-speed and high-precision machining with a smaller interpolation period can be achieved. In this sense, the real-time performance is significantly improved.

## 5. Conclusion

In this paper, a real-time NURBS interpolation is proposed under geometric errors, normal kinematic constraints, and drive constraints. The preprocessing process is used to solve the problem that the velocity in NURBS interpolation is difficult to deal with under the condition of multiple constraints. The comparison and simulation analysis of the proposed RNIC method and the AFI method show that the proposed method has greatly improved in terms of velocity smoothness, acceleration, and jerk continuity. The simulation results show that the proposed algorithm is effective and greatly improves the real-time performance of the NURBS interpolator.

## Figures and Tables

**Table 1:** Forward scanning algorithm.

Forward scanning algorithm
Input: {*u*_*s*_^*segi*^, *v*_*s*_^*i*^, *l*_*i*_^*seg*^, *u*_*e*_^*segi*^, *v*_*e*_^*i*^}, a, J, N
Output: {*u*_*s*_^*segi*^, *v*_*s*_^*i*^, *l*_*i*_^*seg*^, *V*_max_, *u*_*e*_^*segi*^, *v*_*e*_^*i*^}.
1: Initialize: Set i = 0
2: While i < N
3: Calculate *S*_acc_^req^(*v*_*s*_^*i*^, *v*_*F*_), *S*_dec_^req^(*v*_*F*_, *v*_*e*_^*i*^), *S*_acc/dec_^min^(*v*_*s*_^*i*^, *v*_*e*_^*i*^).
4: If *l*_*i*_^*seg*^ ≥ *S*_acc/dec_^min^(*v*_*s*_^*i*^, *v*_*e*_^*i*^).
5: Set *V*_max_=*v*_*F*_.
6: else
7: *v*_*s*_^*i*^=*v*_*e*_^*i*^=min(*v*_*s*_^*i*^, *v*_*e*_^*i*^).
8: Calculate *V*_max−*i*_^act^ to satisfy S(*v*_*s*_^*i*^, *V*_max−*i*_^act^)=*l*_*i*_^*se*g^/2.
9: Set *V*_max_=*V*_max−*i*_^act^.
10: *i* ++

**Table 2:** Backward scanning algorithm.

Backward scanning algorithm
Input: {*u*_*s*_^*segi*^, *v*_*s*_^*i*^, *l*_*i*_^*seg*^, *V*_max_, *u*_*e*_^*segi*^, *v*_*e*_^*i*^}, N
Output: {*u*_*s*_^*segi*^, *v*_*s*_^*i*^, *V*_max_, *u*_dec_, *u*_*e*_^*segi*^, *v*_*e*_^*i*^}.
1: Initialize: Set *i* = 0
2: While *i* < *N*
3: If *V*_max_==*v*_*F*_.
4: Obtained *u*_dec_^*i*^ according to reference 26
5: else if *V*_max_ < *v*_*F*_.
6: *s*_dec_=*l*_*seg*_^*i*^/2.
7: Obtained *u*_dec_^*i*^.
8: *i*++

**Table 3:** The parameters of the simulation.

Parameters	Symbol	Unit
Command feedrate	*v*_max_	0.2 mm/ms
Normal acceleration	*a*_n_	0.001 mm/ms^2^
Normal jerk	*j*_n_	0.0001 mm/ms^3^
Tangential acceleration	*A*_*t*_	0.001 mm/ms^2^
Tangential jerk	*J*_*t*_	0.0001 mm/ms^3^
Max chord error	*δ*_max_	0.001 mm
Max contour error	*ɛ*_max_	0.05 mm

**Table 4:** The parameters of the butterfly-shaped curve.

Parameters	Values
Control points	{54.493, 52.139}, {55.507, 52.139}, {56.082, 49.615},{56.780, 44.971}, {69.575, 51.358}, {77.786, 58.573},{90.526, 67.081}, {105.973, 63.801}, {100.400, 47.326},{94.567, 39.913}, {92.369, 30.485}, {83.440, 33.757},
	{91.892, 28.509}, {89.444, 20.393}, {83.218, 15.446},{87.621, 4.830}, {80.945, 9.267}, {79.834, 14.535},{76.074, 8.522}, {70.183, 12.550}, {64.171, 16.865},{59.993, 22.122}, {55.680, 36.359}, {56.925, 24.995},
	{59.765, 19.828}, {54.493, 14.940}, {49.220, 19.828},{52.060, 24.994}, {53.305, 36.359}, {48.992, 22.122},{44.814, 16.865}, {38.802, 12.551}, {32.911, 8.521},{29.152, 14.535}, {28.040, 9.267}, {21.364, 4.830},
	{25.768, 15.447}, {19.539, 20.391}, {17.097, 28.512},{25.537, 33.750}, {16.602, 30.496}, {14.199, 39.803},{8.668, 47.408}, {3.000, 63.794}, {18.465, 67.084},{31.197, 58.572}, {39.411, 51.358}, {52.204, 44.971},
	{52.904, 49.614}, {53.478, 52.139}, {54.493, 52.139}
Weights	{0, 0, 0, 0, 0.0083, 0.0150, 0.0361, 0.0855, 0.1293, 0.1509, 0.1931, 0.2273, 0.2435, 0.2561,0.2692, 0.2889, 0.3170, 0.3316, 0.3482, 0.3553, 0.3649, 0.3837, 0.4005, 0.4269, 0.4510, 0.4660, 0.4891, 0.5000,0.5109, 0.5340, 0.5489, 0.5731, 0.5994, 0.6163, 0.6351, 0.6447, 0.6518, 0.6683, 0.6830, 0.7111, 0.7307, .7439,0.7565, 0.7729, 0.8069, 0.8491, 0.8707, 0.9145, 0.9639, 0.9850, 0.9917, 1.0, 1.0, 1.0, 1.0};
Knot vector	{1.0, 1.0, 1.0, 1.2, 1.0, 1.0, 1.0, 1.0, 1.0, 1.0, 1.0, 2.0, 1.0, 1.0, 5.0, 3.0, 1.0, 1.1, 1.0, 1.0, 1.0, 1.0, 1.0, 1.0, 1.0, 1.0, 1.0, 1.0, 1.0, 1.0, 1.0, 1.0, 1.0, 1.1, 1.0, 3.0, 5.0, 1.0, 1.0, 2.0, 1.0, 1.0, 1.0, 1.0, 1.0, 1.0, 1.0, 1.2, 1.0, 1.0, 1.0};

**Table 5:** Segments information of the butterfly curve.

Number	U_start	U_end	V_start	V_end	Required_arc length	Acture_arclength	Complete S curve profile
Segment1	0.001	0.0169	0.060443	0.071035	38.307739	6.10257	NO
Segment2	0.0169	0.151502	0.071035	0.136423	31.208744	50.677979	YES
Segment3	0.151502	0.237903	0.136423	0.122992	26.428003	33.198769	YES
Segment4	0.237903	0.256303	0.122992	0.009457	35.054008	7.142123	NO
Segment5	0.256303	0.2753	0.009457	0.142006	32.629776	7.737445	NO
Segment6	0.2753	0.306796	0.142006	0.104414	27.698153	13.756968	NO
Segment7	0.306796	0.336292	0.104414	0.036195	36.596878	8.730752	NO
Segment8	0.336292	0.35529	0.036195	0.03501	41.08812	9.29499	NO
Segment9	0.35529	0.368188	0.03501	0.113086	35.733379	4.824294	NO
Segment10	0.368188	0.449777	0.113086	0.014613	36.137478	31.123468	NO
Segment11	0.449777	0.499971	0.014613	0.091119	38.27055	18.485075	NO
Segment12	0.499971	0.550179	0.091119	0.014629	38.270397	18.501719	NO
Segment13	0.550179	0.631792	0.014629	0.113006	36.145996	31.115427	NO
Segment14	0.631792	0.644695	0.113006	0.035021	35.741718	4.826924	NO
Segment15	0.644695	0.663698	0.035021	0.036196	41.087765	9.303009	NO
Segment16	0.663698	0.693103	0.036196	0.104468	36.591499	8.716472	NO
Segment17	0.693103	0.724608	0.104468	0.141766	27.725693	13.74367	NO
Segment18	0.724608	0.743611	0.141766	0.009517	32.662388	7.756817	NO
Segment19	0.743611	0.762214	0.009517	0.125237	34.786385	7.203535	NO
Segment20	0.762214	0.848428	0.125237	0.135905	26.228525	33.126568	YES
Segment21	0.848428	0.983151	0.135905	0.071057	31.275175	50.711964	YES
Segment22	0.983151	1.0	0.071057	0	39.830734	6.435459	NO

**Table 6:** The comparison of simulation results of the two methods.

Method	Start/end speed	Percentage of scheduling max tan-acc to maximum tan-acc (%)	Percentage of scheduling max tan-jerk to maximum tan-jerk (%)
AFI	2 mm/ms	760	38200
RNIC (proposed)	0 mm/ms	100	100

## Data Availability

The data used to support the findings of this study are available from the corresponding author upon request.
